# PLCγ1 deficiency in chondrocytes accelerates the age‐related changes in articular cartilage and subchondral bone

**DOI:** 10.1111/jcmm.70027

**Published:** 2024-08-19

**Authors:** Qiubo Zhao, Xiaolei Chen, Ning Qu, Jinhua Qiu, Bing Zhang, Chun Xia

**Affiliations:** ^1^ Department of Joint Surgery & Sports Medicine Zhongshan Hospital of Xiamen University, School of Medicine, Xiamen University Xiamen Fujian China; ^2^ School of Medicine Xiamen University Xiamen Fujian China

**Keywords:** abnormalities in subchondral bone, ageing in articular cartilage, Col2a1‐CreERT mice, d‐gal treatment, Plcg1^flox/flox^, PLCγ1, senescence in chondrocytes

## Abstract

Ageing is the most prominent risk for osteoarthritis (OA) development. This study aimed to investigate the role of phosphoinositide‐specific phospholipase Cγ (PLCγ) 1, previously linked to OA progression, in regulating age‐related changes in articular cartilage and subchondral bone. d‐galactose (d‐Gal) was employed to treat chondrocytes from rats and mice or injected intraperitoneally into C57BL/6 mice. RTCA, qPCR, Western blot and immunohistochemistry assays were used to evaluate cell proliferation, matrix synthesis, senescence genes and senescence‐associated secretory phenotype, along with PLCγ1 expression. Subchondral bone morphology was assessed through micro‐CT. In mice with chondrocyte‐specific Plcg1 deficiency (Plcg1^flox/flox^; Col2a1‐CreERT), articular cartilage and subchondral bone were examined over different survival periods. Our results showed that d‐Gal induced chondrocyte senescence, expedited articular cartilage ageing and caused subchondral bone abnormalities. In d‐Gal‐induced chondrocytes, diminished PLCγ1 expression was observed, and its further inhibition by U73122 exacerbated chondrocyte senescence. Plcg1^flox/flox^; Col2a1‐CreERT mice exhibited more pronounced age‐related changes in articular cartilage and subchondral bone compared to Plcg1^flox/flox^ mice. Therefore, not only does d‐Gal induce senescence in chondrocytes and age‐related changes in articular cartilage and subchondral bone, as well as diminished PLCγ1 expression, but PLCγ1 deficiency in chondrocytes may also accelerate age‐related changes in articular cartilage and subchondral bone. PLCγ1 may be a promising therapeutic target for mitigating age‐related changes in joint tissue.

## 1 INTRODUCTION

Ageing is a risk factor for various chronic diseases, including diabetes, Alzheimer's disease and atherosclerosis. Advancing ageing is also the most prominent risk factor of osteoarthritis (OA). OA is characterised by degradation in joint integrity, including cartilage, subchondral bone and synovium. Various age‐related proinflammatory factors and signalling molecules contribute to OA.^1^, ^2^ For instance, levels of IL‐6 in the systemic circulation that increase with age are associated with the risk of OA progressio.^3^ The age‐related loss of Smad2/3 signalling could be considered a normal part of the ageing process, but it could also make articular cartilage vulnerable to OA development in later years compared to young individuals.^2^ On the other hand, pro‐inflammatory factors and extracellular factors that are overexpressed in OA pathogenesis may play essential roles in the ageing process. Matrix metalloproteinases (MMPs) responsible for extracellular matrix degradation are an important subset of senescence‐associated secretory phenotypes (SASP).^4^ IL‐1 (IL‐1α and IL‐1β) is both a proinflammatory factor and one of the major components of SASP.^4^, ^5^ Therefore, despite ageing and OA being interconnected processes, identical signalling molecules can have distinct roles in each process. Phosphoinositide‐specific phospholipase Cγ (PLCγ) is a key signal molecule involved in various life processes, such as cell metabolism, cell cycle and extracellular matrix synthesis, which contains two isoforms (PLCγ1 and PLCγ2). Biswas et al.^6^ find that PLCγ2 increases in the case of OA. Daisuke et al.^7^ report that PLCγ mediated FGFR3‐induced STAT1 activation and this signalling cascade involved in the induction of apoptosis in the chondrogenic cell line ATDC5. Lu et al.^8^'s study shows that chondrocyte migration affects tissue‐engineered cartilage integration by activating the signal transduction pathways involving Src, PLCγ1 and ERK1/2. Our previous studies demonstrated that PLCγ1 inhibition has a chondrocyte protective effect on IL‐1β‐treated or OA chondrocytes.^9^, ^10^, ^11^ However, it is unclear whether PLCγ1 expression is linked to the age‐related changes in articular cartilage and subchondral bone.

To elucidate the role of PLCγ1 in senescent chondrocytes and ageing cartilage, we first investigated whether d‐galactose (d‐Gal) was utilised to induce senescence in mouse chondrocytes, as well as ageing in articular cartilage and abnormalities in subchondral bone, both in vitro and in vivo. Subsequently, we explored the relationship between PLCγ1 and age‐related changes in mouse articular cartilage and subchondral bone through in vitro and in vivo research. Lastly, we evaluated the age‐related changes in articular cartilage and subchondral bone in Plcg1^flox/flox^; Col2a1‐CreERT mice. Our results indicated that d‐Gal could potentially induce senescence in chondrocytes, ageing in articular cartilage and abnormalities in subchondral bone, as well as diminished PLCγ1 expression. Furthermore, PLCγ1 deficiency in chondrocytes accelerates the age‐related changes in articular cartilage and subchondral bone.

## 2 MATERIALS AND METHODS

### 2.1 Chondrocyte isolation and culture

Approved by the Committee on the Ethics of Animal Experiments of Xiamen University (XMULAC20190130), primary chondrocytes were obtained from the epiphyseal and articular cartilage of the extremities in neonatal male Sprague Dawley (SD) rats or C57BL/6J mice (within 24–72 h after birth). Primary chondrocytes were cultured in Dulbecco's modified Eagle medium (DMEM) containing 10% foetal bovine serum (FBS) and 1% penicillin/streptomycin to 80% confluence and plated in 60‐mm cell culture dishes as previously described.^12^ Passages 1–2 chondrocytes were for the subsequent experiments.

### 2.2 Animals

Sixteen 8‐week‐old male C57BL/6 mice were procured from the Laboratory Animal Center of Xiamen University. Mice were randomly assigned to two groups, the Control and d‐Gal group. The d‐Gal group received intraperitoneal injections of d‐galactose (400 mg/Kg/d) daily, while the Control group was injected with the same volume of normal saline. Until 8 week, mice were sacrificed for the following examination.

Plcg1^flox/flox^ mice were purchased from Cyagen Biosciences Inc. with C57BL/6 genetic background (Quote: TOS190329VZ1). Col2a1‐CreERT mice were donated by Professor Ren Xu at Xiamen University School of Medicine. Through continuous hybridization and identification, Plcg1^flox/flox^; Col2a1‐CreERT mice obtained were further divided into two groups, Cre‐positive (Plcg1^flox/flox^; Col2a1‐CreERT mice) and Cre‐negative group (Plcg1^flox/flox^ mice). To initiate Plcg1 knockout, Plcg1^flox/flox^; Col2a1‐CreERT mice were intraperitoneally injected with tamoxifen (20 mg/Kg/d) for 5 days, while Plcg1^flox/flox^ mice were intraperitoneally injected with the same volume of corn oil. The Committee on Ethics of Animal Experimentation at Xiamen University approved all animal procedures (XMULAC20190130, 20230215).

### 2.3 Senescence‐associated β‐galactosidase (SA‐β‐gal) staining

In brief, chondrocytes were washed three times with PBS, fixed in a 10% paraformaldehyde solution for 15 min at room temperature and incubated overnight at 37°C in darkness with the working solution containing 0.05 mg/mL 5‐bromo‐4‐chloro‐3‐indolyl‐β‐d‐galactopyranoside (X‐gal) (C0602, Beyotime, China).

### 2.4 Real‐time cellular analysis (RTCA)

According to the manufacturer instruction of RTCA iCELLigence (ACEA Biosciens. Inc., San Diego, CA92121, USA), 1 × 10^3^ chondrocytes were seeded in two 8‐well plates with an integrated microelectronic sensor assay for 120 h. The cell index (CI) was monitored every hour by the iCELLigence system and calculated for each E‐plate well using RTCA software1.2 (Roche Diagnostics, Meylan, France).^13^ The graphs were real‐time generated outputs from the iCELLigence system.^14^

### 2.5 Western blot analysis

As described previously,^15^ total proteins were extracted, and the concentrations were measured using a BCA assay. Samples were subjected to SDS‐PAGE (8%–12%) and transferred to PVDF membranes (GE Healthcare, Hertfordshire, UK). The membranes were incubated with primary antibodies at 4°C overnight (Table 1), followed by incubation with the corresponding secondary antibodies at room temperature for 1 h. Specific antibody reactivity was detected by enhanced chemiluminescence (Merck Millipore, Billerica, MA, USA). GAPDH was used as an internal control.

**TABLE 1:** Information of antibodies.

Antibody	Manufacturer	Ratio of dilution	Time
P16	Abcam ab189034	1:100 (IHC) 1:1000 (WB)	4°C overnight
P16	Abcam ab54210	1:1000 (WB)	4°C overnight
P21	Proteintech Cat No.28248‐1‐AP	1:100 (IHC) 1:1000 (WB)	4°C overnight
P53	Proteintech Cat No.10442‐1‐AP	1:100 (IHC) 1:1000 (WB)	4°C overnight
MMP13	Abcam ab39012	1:1000 (WB)	4°C overnight
ADAMTS5	Abcam ab41037	1:1000 (WB)	4°C overnight
COL2	Abcam ab34712	1:50 (IHC) 1:1000 (WB)	4°C overnight
COL2	Abcam ab188570	1:1000 (WB)	4°C overnight
Agg	Proteintech Cat No. 13880‐1‐AP	1:100 (IHC) 1:1000 (WB)	4°C overnight
PLC‐γ1	Cell Signalling Technology #5690	1:100 (IHC) 1:1000 (WB)	4°C overnight
p‐PLC‐γ1 (Tyr783)	Cell Signalling Technology #2821	1:100 (IHC) 1:1000 (WB)	4°C overnight
GAPDH	Proteintech Cat No. 10494‐1‐AP	1:2000 (WB)	4°C overnight
anti‐Mouse‐IgG (H + L)HRP	Proteintech SA00001‐1	1:25000	Room temperature 1 h
anti‐Rabbit‐IgG (H + L)HRP	Proteintech SA00001‐2	1:25000	Room temperature 1 h

### 2.6 Real‐time quantitative polymerase chain reaction (qPCR)

Total RNA in different cells was extracted as described in the manufacturer's instruction of BioFlux Simply P Total RNA Extraction Kit (BIOMARS, Beijing, China). After cDNA was synthesised with 2 μg of total RNA at 37°C for 15 min using a 5 × EvoM‐MLV RT Master Mix (AG, Guangzhou, China), qPCR was performed using a Roche LightCycler 96 (Roche, Switzerland) with a MonAmpTM ChemoHS qPCR Mix (Monadbiotech, Wuhan, China), as previously described.^16^ SDS software v2.1. was employed to quantitative gene expression relevant to the GAPDH housekeeping gene. The primers used for quantitative PCR were listed in Table 2.

**TABLE 2:** Primers of qPCR.

Gene name	Primer sequence (5′–3′)
Plcg1 (R) Gene ID: 25738 NM_013187.2	Forward primer: AGTTGGTGGCATAGGAAGCT Reverse primer: TGTGCTGTCTTCCTCCTCAG
Plcg1 (M) Gene ID: 18803 NM_021280.3	Forward primer: CAACCAGCTCAGGAGGAAGA Reverse primer: TTCATCCTCATTGCCCTGGT
SIRT1 (R) Gene ID: 309757 NM_001372090.1	Forward primer: ATGAAGTATGACAAAGATGAAGT Reverse primer: GTAGATGAGGCAGAGGTT
SIRT1 (M) Gene ID: 93759 AY377984.1	Forward primer: TGCCATCATGAAGCCAGAGA Reverse primer: AACATCGCAGTCTCCAAGGA
SIRT6 (R) Gene ID: 299638 NM_001031649.1	Forward primer: GCTGGAGCCCAAGGAGGAATC Reverse primer: AGTAACAAAGTGAGACCACGAGAG
SIRT6 (M) Gene ID: 50721 BC052763.1	Forward primer: GACACCACCTTCGAGAATGC Reverse primer: AAACATGTTTCCGTGCAGCT
P16 INK4a (R) Gene ID: 25163 NM_031550.2	Forward primer: CGATACAGGTGATGATGATG Reverse primer: TACTACCAGAGTGTCTAGGA
P16 INK4a (M) Gene ID: 12578 BC058190.1	Forward primer: CTTCTGCTCAACTACGGTGC Reverse primer: GCACGATGTCTTGATGTCCC
P21 (R) Gene ID: 114851 NM_080782.4	Forward primer: AACCTGTCTCTTGGATATTCT Reverse primer: GGACCATAGGCACATCTT
P21 (M) Gene ID: 12575 BC002043.1	Forward primer: ACAAGAGGCCCAGTACTTCC Reverse primer: GGGCACTTCAGGGTTTTCTC
P53 (R) Gene ID: 24842 NM_030989.3	Forward primer: AATGGGTTGGTAGTTGCT Reverse primer: CAGAGTGGAGGAAATGGG
P53 (M) Gene ID: 22059 U59757.1	Forward primer: CTCCCCAGCATCTTATCCGG Reverse primer: ATGGTAAGGATAGGTCGGCG
Cav1 (R) Gene ID: 25404 BC161826.1	Forward primer:TCTACAAGCCCAACAACAAGGCC Reverse primer:TGCACTGAATCTCAATCAGGAAGC
Cav1 (M) Gene ID: 12389 NM_007616.5	Forward primer: AGGCAGGGAAGAGTACCAAC Reverse primer: AGAGTGAGGACAGCAACCAA
MMP13 (R) Gene ID: 171052 NM_133530.1	Forward primer: CCCTGGAGCCCTGATGTTT Reverse primer: CTCTGGTGTTTTGGGGTGCT
MMP13 (M) Gene ID: 17386 NM_008607.2	Forward primer: ACCAGAGAAGTGTGACCCAG Reverse primer: AAGGTCACGGGATGGATGTT
IL‐8 (R) Gene ID:	Forward primer: CCTTGGTCTTCCTGCTTGA Reverse primer: ATCGTTGTTCCCATCCACAT
IL‐8 (M) Gene ID: 20309 NM_011339.2	Forward primer: ATGCCTCTCCATTTCCTGCT Reverse primer: CATGGGGAAAGAGGCTCTGA
GAPDH (R) Gene ID: 24383 XM_017592435.1	Forward primer: GGCACCCAGCACAATGAA Reverse primer: GCCGATCCACACGGAGTACT
GAPDH (M) Gene ID: 14433 GU214026.1	Forward primer: CAACTCCCACTCTTCCACCT Reverse primer: GAGTTGGGATAGGGCCTCTC

### 2.7 Micro‐computed tomography (micro‐CT) analysis

Samples were carefully removed from soft tissues and fixed with 4% paraformaldehyde for 48 h prior to micro‐CT analysis. Samples were scanned in a micro‐CT scanner Skyscan 1272 (Bruker, Belgium), as described previously.^11^, ^17^ Images were scanned at an X‐ray source voltage of 80 kV and a resolution of 13.2 μm using Al filters of 0.5 mm thickness to capture the best X‐ray projections. Images were then reconstructed using the NRecon program (Version: 1.7.4.2). Various parameters were calculated using CTAn software (Version: 1.17.7.2), and 3D images of each group were reconstructed using CTVox software (Version: 3.3.0r1403).

### 2.8 Histological and Immunohistochemistry assays

The samples were decalcified using 15% EDTA‐2Na for 3 weeks, followed with routine histological preparation. Four‐micrometre‐thick sections were processed for haematoxylin and eosin (H.E.) and Safranin O/Fast Green stainings, following evaluating the thickness of articular cartilage using the ratio of HC layer to the combined CC with HC layer (HC/CC + HC) and OARSI Score.^11^ Immunohistochemical staining was performed according to the manufacturer's instructions (MAXIN, BIO, Fuzhou, Fujian Province, China). Briefly, the sections were incubated with primary antibodies at 4°C overnight (Table 1), followed by incubation with HRP‐conjugated secondary antibodies for 10 minutes. Diaminobenzidine (DAB) was used to visualise the immunohistochemical reaction followed by being counterstained with haematoxylin. As previously described,^11^, ^18^ dark brown cells and areas were measured using Image J and Image‐Pro Plus 6.0 Softwares, respectively, followed by analysis using GraphPad Prism version 5 (GraphPad Software, Inc., San Diego, CA, USA).

### 2.9 Statistical analysis

Statistical analysis was conducted using SPSS 19.0 software (SPSS, Chicago, IL), and the data were presented as the mean ± SD. The statistical significance between groups was analysed using the *t*‐test or one‐way analysis of variance (ANOVA) followed by Dunnett's or Tukey's post hoc test using GraphPad Prism version 6. A *p*‐value less than 0.05 was considered statistically significant.

## 3 RESULTS

### 3.1 d‐Gal induced senescence in chondrocytes in vitro and age‐related changes in articular cartilage and subchondral bone in vivo

**FIGURE 1:**
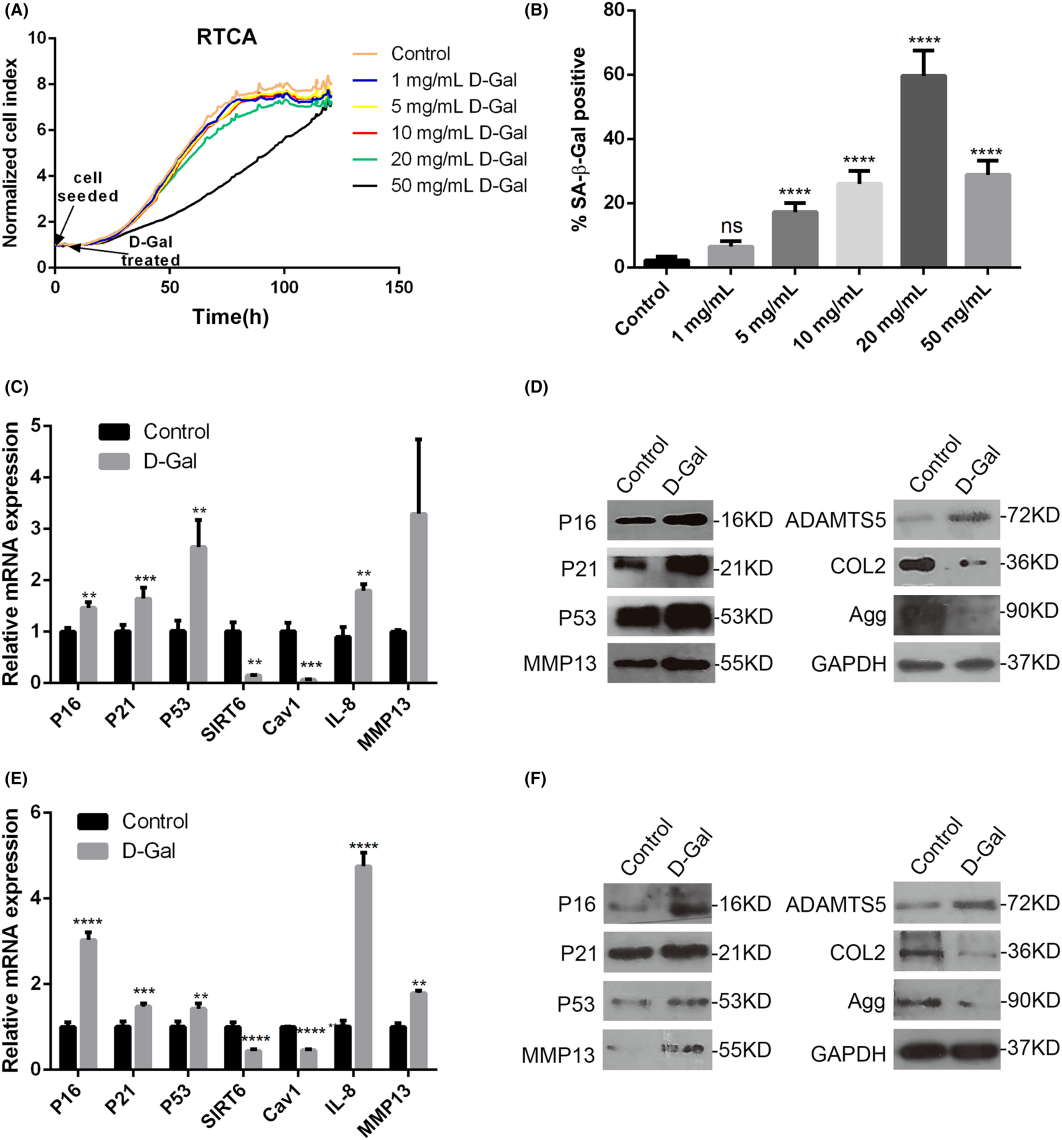
Inductive effect of d‐Gal on chondrocyte senescence. (A, B) Rat chondrocytes were treated with different concentrations of d‐Gal for 120 h or 72 h, followed by RTCA (A) and SA‐β‐gal staining (B), respectively. (C, D) Rat chondrocytes were treated with 20 mg/mL d‐Gal for 72 h, followed by qPCR and Western blot analyses. (E, F) Mouse chondrocytes were treated with 20 mg/mL d‐Gal for 72 h, followed by qPCR and Western blot analysis. Data were representative of three independent experiments and reported as mean ± SD (**p < 0.01, ***p < 0.001, ****p < 0.0001).

Because of its convenience, minimal side effects and high survival rate of model animals, d‐Gal is widely used to be an effective inducer for establishing senescent cells or ageing animal models of main organs.^19^, ^20^, ^21^, ^22^, ^23^ However, it was not confirmed whether d‐Gal was utilised to induce senescent chondrocytes or ageing animal models of articular cartilage and subchondral bone. Here, rat primary chondrocytes were treated with d‐Gal at various concentrations (1, 5, 10, 20 and 50 mg/mL) for an incubation period of 120 h. The proliferation rates of these chondrocytes were quantitatively evaluated using Real‐Time Cell Analysis (RTCA). Compared with the Control group, there was no significant difference in chondrocyte proliferation rates across the majority of d‐Gal concentrations (Figure 1A). However, a discernible reduction in proliferation rate was observed in chondrocytes exposed to 50 mg/mL d‐Gal, compared with other treatment groups and the Control group, prior to the completion of the 120‐hour exposure period (Figure 1A). Furthermore, the percentage of SA‐β‐Gal positive cells significantly rose with increasing concentrations of d‐Gal, with the exception at 50 mg/mL d‐Gal. At this concentration, both the cell density and number of SA‐β‐Gal positive cells were significantly lower than that at 20 mg/mL (Figure 1B; Figure S1A). Thus, d‐Gal at 20 mg/mL was employed in the subsequent in vitro investigations. The results of qPCR in Figure 1C showed that in d‐Gal‐treated rat chondrocytes, mRNA expression levels of senescent genes, including P16, P21 and P53, as well as SASP genes, including IL‐8 and MMP13, were significantly higher, while the longevity gene SIRT6 and the senescence‐related gene Cav1 decreased substantially (compared to the Control group). Figure 1D showed that in d‐Gal‐treated chondrocytes, the protein expressions of P16, P21, P53, MMP13 and ADAMTS5 increased, whereas the protein expressions of matrix major components collagen type II (COL2) and Aggrecan (Agg) decreased (compared to the Control group). Figure 1E,F and Figure S1B showed similar results in d‐Gal‐treated mouse chondrocytes.

**FIGURE 2:**
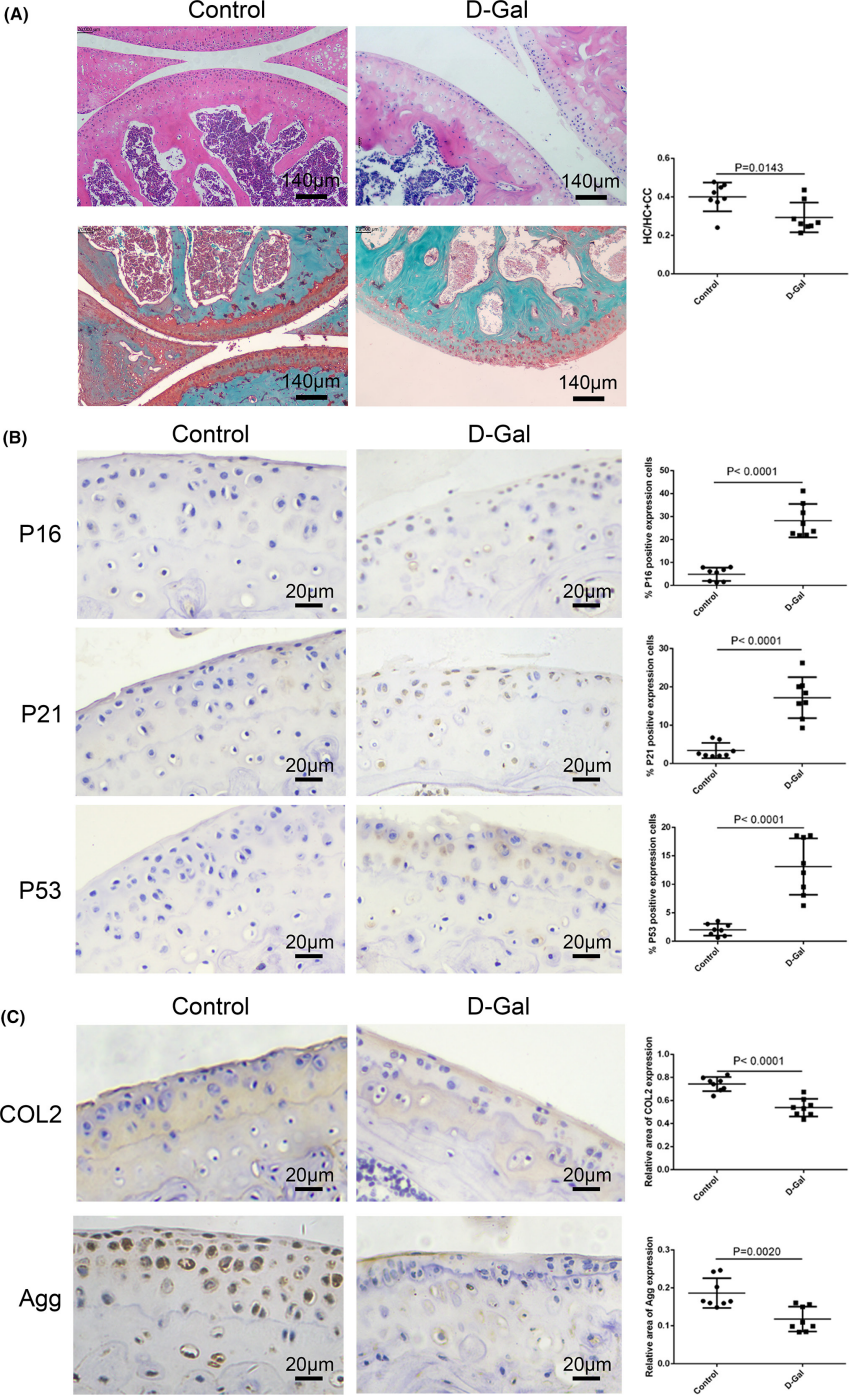
Inductive effect of d‐Gal on articular cartilage ageing and subchondral bone abnormalities. (A) Representative H.E. and Safranin O/Fast Green staining images of articular cartilage in control and d‐Gal‐injected mice, accompanied with the statistical graph of HC/HC + CC ratio (*n* = 8, Bar = 140 μm). (B) Representative immunohistochemical staining images for P16, P21 and P53 in articular cartilage of control and d‐Gal‐injected mice, accompanied by their statistical graphs (*n* = 8, Bar = 20 μm). (C) Representative immunohistochemical staining images for COL2 and Agg in articular cartilage of control and d‐Gal‐injected mice, accompanied by their statistical graphs (*n* = 8, Bar = 20 μm). Data were reported as mean ± SD and *p* value was indicated on the graphs.

**FIGURE 3:**
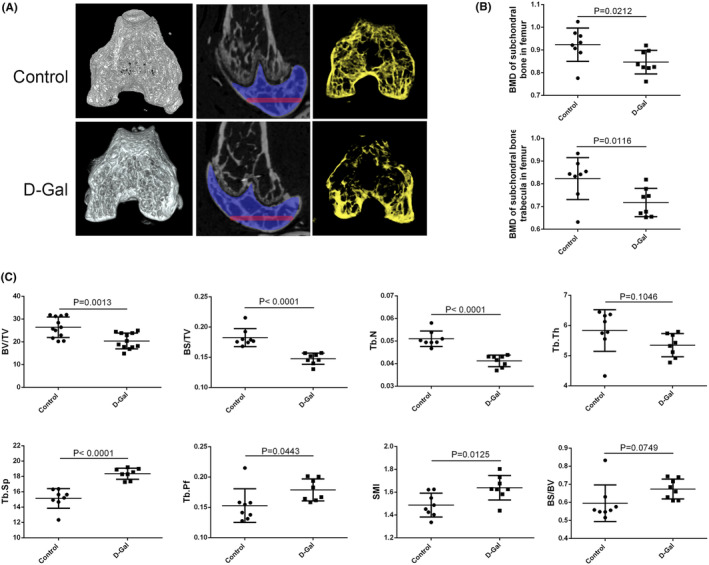
Effect of d‐Gal injection on the femur subchondral bone. (A) Representative three‐ or two‐dimensional image of micro‐CT analysis in the femur subchondral bone. (B) Quantitative analysis of bone mineral density (BMD) in subchondral bone and subchondral bone trabecula by micro‐CT analysis. (C) Quantitative analysis of structural parameters by micro‐CT analysis: Bone volume/total volume (BV/TV), bone surface /total volume (BS/TV), trabecular number (Tb. N), trabecular thickness (Tb. Th), trabecular separation (Tb. Sp), trabecular pattern factor (Tb. Pf), structural model index (SMI) and bone surface/bone volume (BS/BV). Data were reported as mean ± SD and *p* value was indicated on the graphs.

In addition, following 8‐week d‐Gal intraperitoneal injection in C57BL/6 mice (Figure S2A), the cartilage surface in the knee joint showed minor damage, as evidenced by a significantly reduced ratio of hyaline cartilage (HC) layer to the combined calcified cartilage (CC) with HC layer (HC/HC + CC) compared with the Control group (Figure 2A; Figure S2B). Concurrently, there was no significant difference in the volume of proteoglycans and OARSI score between d‐Gal and control groups (Figure 2A; Figure S2C). Meanwhile, the d‐Gal group had more P16, P21 and P53 positive cells in articular cartilage than that in the Control group (Figure 2B), while the relative area of COL2 and Agg expressions was lower than that in the Control group (Figure 2C). Additionally, micro‐CT data demonstrated that when compared to the Control group, bone mineral density (BMD) of either the total subchondral bone or subchondral bone trabeculae in the femur was significantly lower in the d‐Gal group (Figure 3A,B). Bone volume (BV)/total volume (TV), bone surface (BS)/total volume (TV), trabecular number (Tb. N) and trabecular thickness (Tb. Th) in the subchondral bone trabeculae significantly decreased in the d‐Gal group, while trabecular space (Tb. Sp), trabecular pattern factor (Tb. Pf) and structural model index (SMI) substantially increased (Figure 3C). Likewise, changes in these parameters in the tibial subchondral bone were similar to those in the femur (Figure S3). As a result of these findings, d‐Gal was capable of inducing senescence in chondrocytes in vitro and age‐related changes in articular cartilage and subchondral bone in vivo.

### 3.2 PLCγ1 was involved in chondrocyte senescence

**FIGURE 4:**
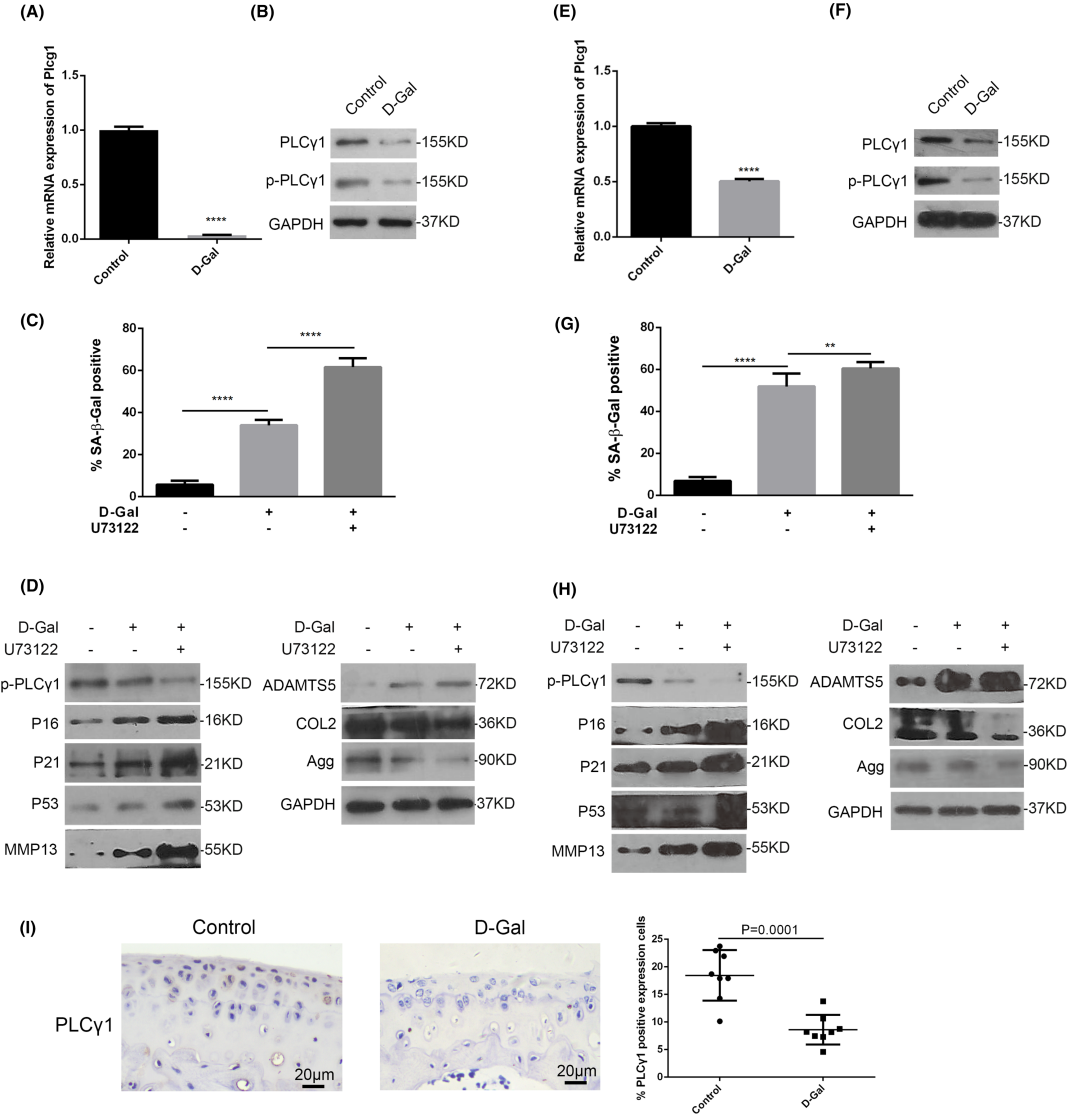
Involvement of PLCγ1 in chondrocyte senescence induced by d‐Gal. (A, B) Rat chondrocytes were treated with 20 mg/mL d‐Gal for 72 h, followed by qPCR (A) and SA‐β‐gal staining (B), respectively. (C, D) Rat chondrocytes were treated with 20 mg/mL d‐Gal for 48 h prior to the co‐treatment with U73122 (2 μM) for 24 h, followed by SA‐β‐gal staining (C) and Western blot analysis (D). (E, F) Mouse chondrocytes were treated with 20 mg/mL d‐Gal for 72 h, followed by qPCR (E) and SA‐β‐gal staining (F), respectively. (G, H) Mouse chondrocytes were treated with 20 mg/mL d‐Gal for 48 h prior to the co‐treatment with U73122 (2 μM) for 24 h, followed by SA‐β‐gal staining (G) and Western blot analysis (H). (I) Representative immunohistochemical staining images for PLCγ1 in articular cartilage of the control and d‐Gal‐injected mice, accompanied by their statistical graph (*n* = 8, Bar = 20 μm). Data were representative of three independent experiments and reported as mean ± SD (**p < 0.01, ****p < 0.0001).

A significant decrease in PLCγ1 mRNA expression was observed in d‐Gal‐treated rat chondrocytes, as well as total PLCγ1 and phospho‐PLCγ1 (p‐PLCγ1) protein expression (Figure 4A,B). Furthermore, the addition of U73122, a substance that blocks the activity of PLCγ1, significantly increased the number of SA‐β‐Gal positive cells in d‐Gal‐treated chondrocytes compared to chondrocytes untreated with U73122 (Figure 4C; Figure S4A), Meanwhile, U73122 aggravated the effect of d‐Gal on the protein expressions of P16, P21, P53, MMP13, ADAMTS5 and COL2 and Agg (Figure 4D). Similar observations were made in mouse chondrocytes (Figure 4E–H; Figure S4B). Additionally, the d‐Gal group had significantly fewer PLCγ1 positive cells in the articular cartilage of C57BL/6 mice intraperitoneally injected with d‐Gal for 8 weeks than that in the Control group (Figure 4I).

**FIGURE 5:**
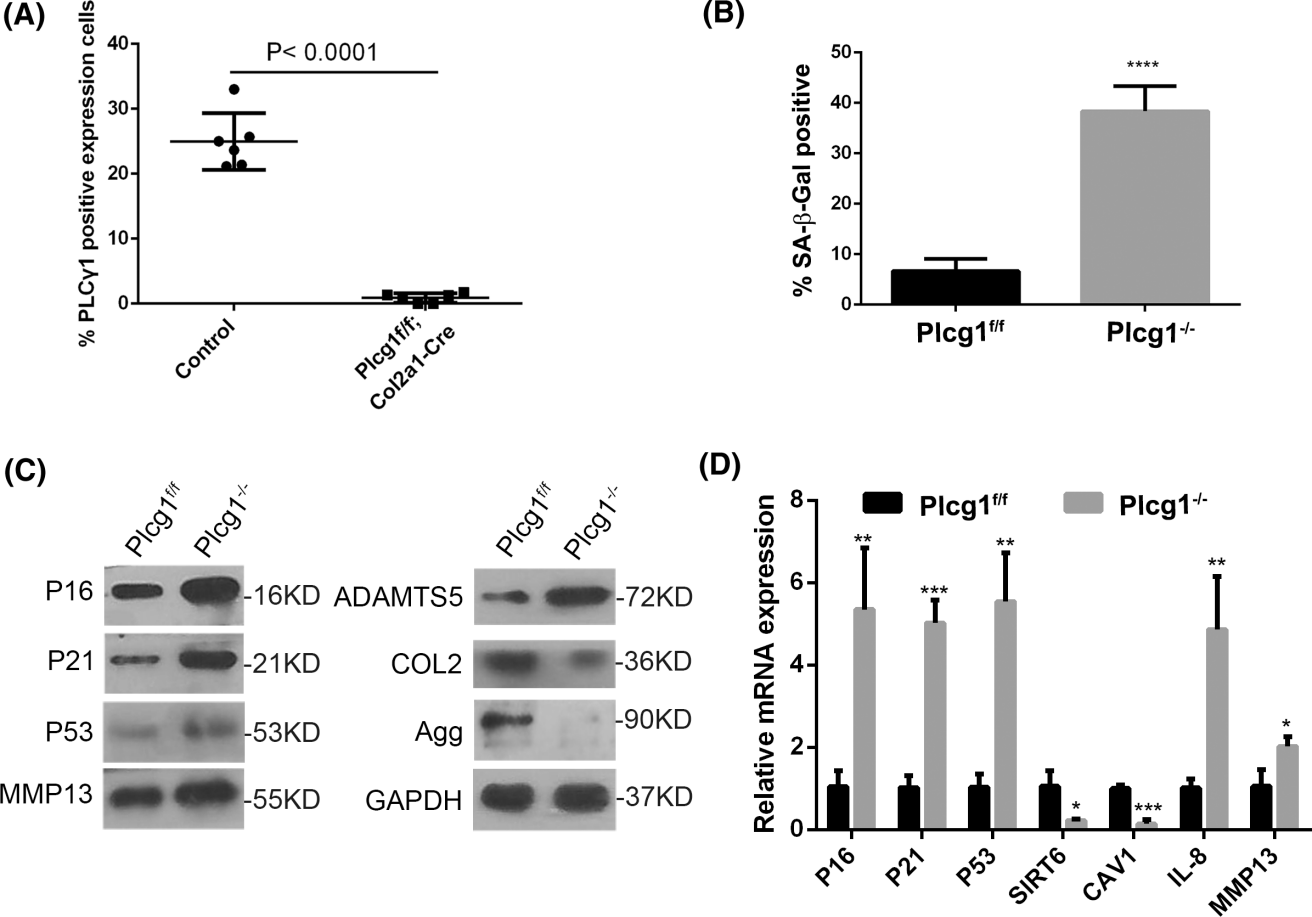
The involvement of PLCγ1 in mouse chondrocyte senescence. (A) Statistical graph of the percentage of PLCγ1 positive expression cells in articular cartilage in Plcg1^flox/flox^; Col2a1‐CreERT mice at 2‐week post‐induction following intraperitoneal injection of tamoxifen or corn oil for 5 days (*n* = 6). (B–D) Chondrocytes isolated from articular cartilage in Plcg1^flox/flox^; Col2a1‐CreERT mice were performed by SA‐β‐gal staining (B), Western blot analysis (C) and qPCR (D). Data were representative of three independent experiments and reported as mean ± SD (*p < 0.05, **p < 0.01, ***p < 0.001, ****p < 0.0001).

In addition, PLCγ1 protein expression level was quantified in articular cartilage of Plcg1^flox/flox^; Col2a1‐CreERT mice at 2‐week post‐induction following intraperitoneal injection of tamoxifen (Figure S5A,B). Figure 5A and Figure S5C showed that PLCγ1 protein expression in articular cartilage of Plcg1^flox/flox^; Col2a1‐CreERT (hereinafter referred to as Plcg1^f/f^; Col2a1‐Cre) mice was lower than that in Plcg1^flox/flox^ (hereinafter referred to as Control) mice. There was a significant increase in the number of SA‐β‐Gal positive chondrocytes derived from Plcg1^f/f^; Col2a1‐Cre mice (hereinafter referred to as Plcg1^−/−^ chondrocytes) for 72 h, compared to chondrocytes derived from Control mice (hereinafter referred to as Plcg1^f/f^ chondrocytes) (Figure 5B). In Plcg1^−/−^ chondrocytes, the protein expression levels of P16, P21, P53, MMP13 and ADAMTS5 increased, while COL2 and Agg decreased (Figure 5C). The mRNA expression levels of senescent genes P16, P21 and P53 and SASP genes, including IL‐8 and MMP13, significantly increased, whereas the genes related to senescence SIRT6 and Cav1 decreased substantially (Figure 5D). Consequently, these findings showed that PLCγ1 was involved in chondrocyte senescence, which negatively impacted the senescence phenotype.

### 3.3 Chondrocyte‐specific Plcg1 deficiency accelerated the ageing process in articular cartilage and subchondral bone

**FIGURE 6:**
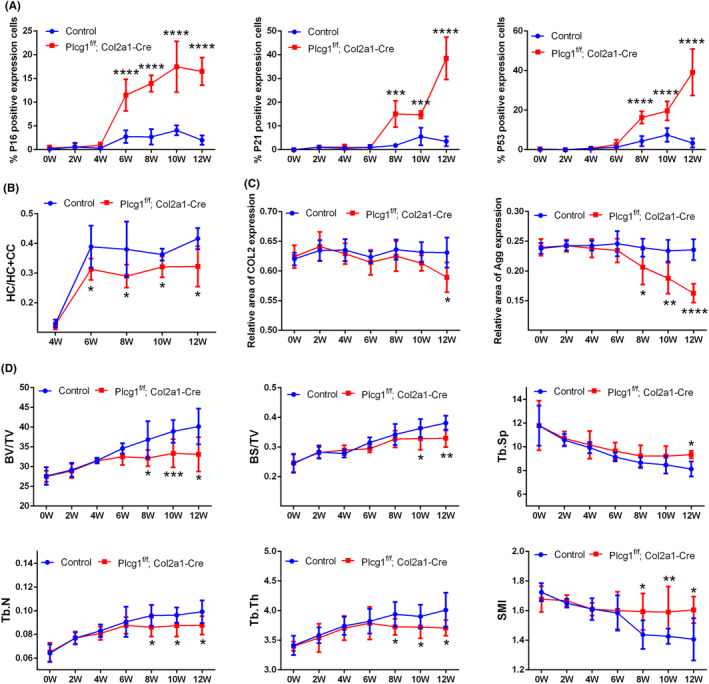
Effect of chondrocyte‐specific PLCγ1 deficiency on the ageing process in articular cartilage and femur subchondral bone. (A) Statistical graphs of the percentage of P16, P21 and P53 positive expression cells in articular cartilage of Plcg1^flox/flox^; Col2a1‐CreERT and Control mice. (B) Statistical graph of HC/IHC + CC ratio in articular cartilage of Plcg1^flox/flox^; Col2a1‐CreERT and Control mice. (C) Statistical graphs of relative expression area of COL2 and Agg in articular cartilage of Plcg1^flox/flox^; Col2a1‐CreERT and Control mice (D). Statistical graphs of the structural parameters in subchondral bone of Plcg1^flox/flox^; Col2a1‐CreERT and Control mice: Bone volume/total volume (BV/TV), bone surface /total volume (BS/TV), trabecular separation (Tb. Sp), trabecular number (Tb. N), trabecular thickness (Tb. Th) and structural model index (SMI). Data were reported as mean ± SD (*p < 0.05, **p < 0.01, ***p < 0.001, ****p < 0.0001).

To study the impact of chondrocyte‐specific Plcg1 deficiency on the ageing process in articular cartilage and subchondral bone, we assessed the thickness of the HC layer and senescence‐ and matrix synthesis‐related biomarkers in articular cartilage and the structural parameters in subchondral bone in Plcg1^flox/flox^; Col2a1‐CreERT mice. Measurements were taken at 0‐, 2‐, 4‐, 6‐, 8‐, 10‐ and 12‐week post‐induction of chondrocyte‐specific Plcg1 deficiency. Because the articular cartilage had not fully developed in groups 0‐, 2‐ or 4‐week post‐induction, there were no differences in the thickness of the HC layer in articular cartilage, as well as P16, P21, P53, COL2 and Agg expression levels and the structural parameters in the subchondral bone between Plcg1^f/f^; Col2a1‐Cre and Control mice (Figures S6 and S7; Figure 6). Beginning 6‐week post‐induction and continuing through 8, 10 and 12 weeks, the percentage of cells expressing P16 in the articular cartilage was considerably higher than that in Control mice, while the percentage of cells expressing P21 and P53 in 8‐, 10‐ and 12‐week Plcg1^f/f^; Col2a1‐Cre mice were considerably higher than that in Control mice of the same age (Figure 6A; Figure S8A). Furthermore, the HC/HC + CC ratio in 6‐, 8‐, 10‐ and 12‐week Plcg1^f/f^; Col2a1‐Cre mice became significantly lower than that in Control mice of the same age (Figure 6B; Figure S8B). Although the area of COL2 expression in articular cartilage was significantly reduced only in 12‐week Plcg1^f/f^; Col2a1‐Cre mice, the areas of Agg expression in 8, 10 and 12 weeks were significantly smaller than that in Control mice of the same age (Figure 6C; Figure S8C). Additionally, beginning at 8‐week post‐induction and continuing through 10 and 12 weeks, the structural parameters in the femur subchondral bone, such as BV/TV, Tb. N and Tb. Th, were significantly lower in Plcg1^f/f^; Col2a1‐Cre mice than that in Control mice of the same age, while BS/TV was lower in 10‐ and 12‐week Plcg1^f/f^; Col2a1‐Cre mice than that in Control mice of the same age (Figure 6C; Figure S9A). SMI was substantially higher in 8‐, 10‐ and 12‐week Plcg1^f/f^; Col2a1‐Cre mice than that in Control mice of the same age, while Tb. Sp was markedly different in only 12‐week Plcg1^f/f^; Col2a1‐Cre mice (Figure 6D; Figure S9A). Similar changes in these parameters were observed in the tibial subchondral bone (Figures S9B & S10). As a result, chondrocyte‐specific Plcg1 deficiency, beginning 6‐week post‐induction and continuing through 8, 10 and 12 weeks, expedited the ageing process in both articular cartilage and subchondral bone, as evidenced by elevated ageing‐related gene protein expression, decreased ratio of HC/HC + CC, lower levels of COL2 and Agg protein expression, and abnormal bone formation parameters.

## 4 DISCUSSION

**FIGURE 7:**
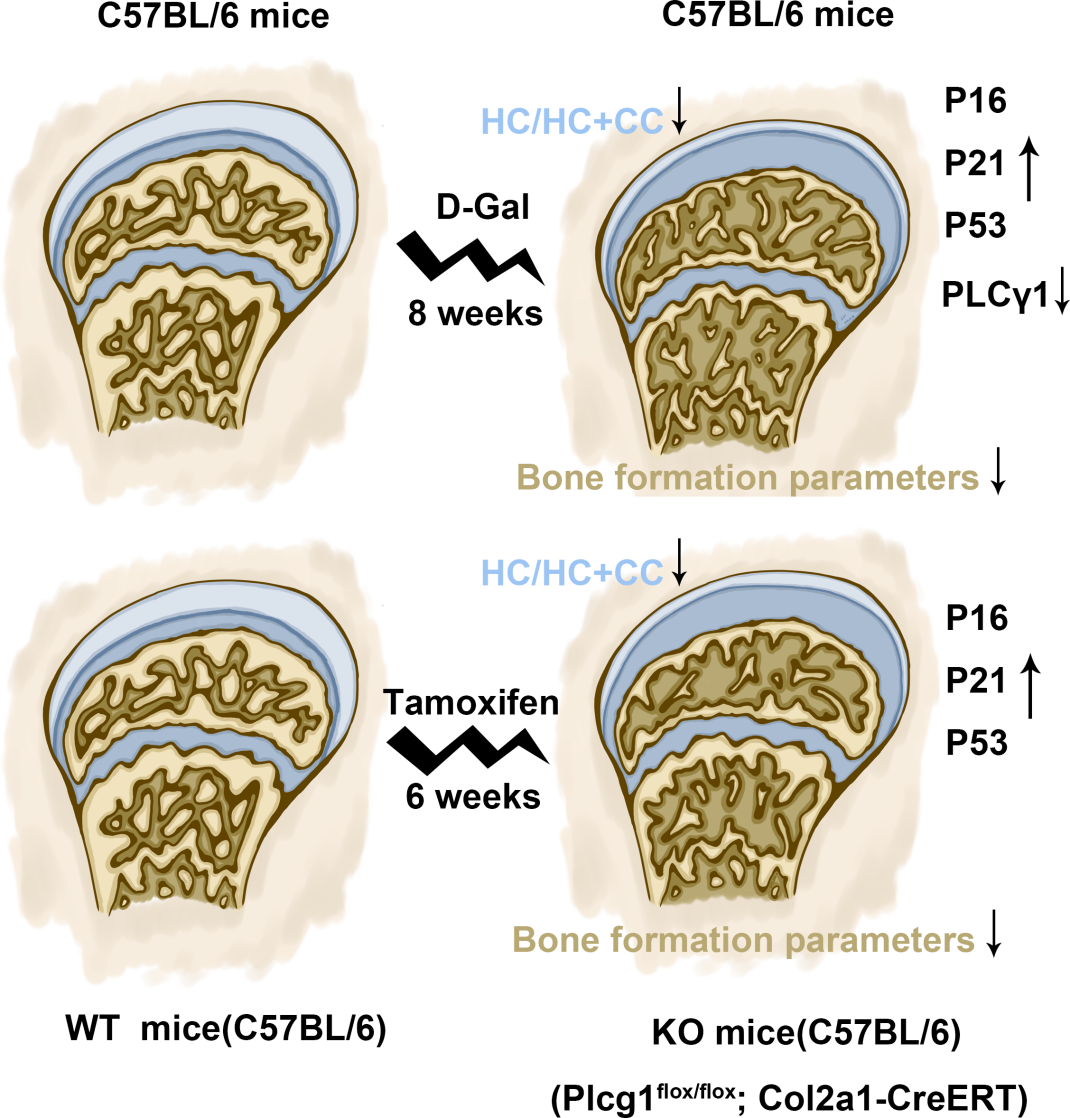
Schematic diagram illustrating the impact of PLCγ1 on the age‐related changes in articular cartilage and subchondral bone.

Our findings demonstrated that d‐Gal reduced PLCγ1 expression level and PLCγ1 inhibitor, U73122, exacerbated the chondrocyte senescence caused by d‐Gal. Moreover, Plcg1^flox/flox^; Col2a1‐CreERT mice exhibited more dramatic age‐related alterations in both articular cartilage and subchondral bone compared to Plcg1^flox/flox^ mice. Especially from 6‐ to 12‐week post‐induction, Plcg1^flox/flox^; Col2a1‐CreERT mice experienced an accelerated ageing process in articular cartilage and subchondral bone compared to Plcg1^flox/flox^ mice of the same age. Therefore, PLCγ1 emerges as a promising therapeutic target for mitigating age‐related changes in joint tissue (Figure 7).

Previous research has addressed the role of PLCγ in the ageing process of neurons. Ageing and a peripheral immune challenge interact to reduce mature brain‐derived neurotrophic factor and activation of TrkB, PLCg1 and ERK in hippocampal synaptoneurosomes.^24^ Deletion of PLCγ1 in GABAergic neurons increases seizure susceptibility in aged mice.^25^ In this study, PLCγ1 expression was diminished in rat and mouse chondrocytes treated with d‐Gal, as well as in the articular cartilage of C57BL/6 mice injected with d‐Gal. Concomitantly, PLCγ1 inhibitor U73122 further intensified the senescent phenotype in chondrocytes induced by d‐Gal, as evidenced by an increase in the number of SA‐β‐Gal positive cells and enhanced senescent phenotype. Furthermore, when compared with Plcg1^f/f^ chondrocytes, the protein expression levels of P16, P21, P53, MMP13 and ADAMTS5 increased, while COL2 and Agg decreased in Plcg1^−/−^ chondrocytes. Simultaneously, the mRNA expression levels of senescent genes P16, P21 and P53 and SASP genes, including IL‐8 and MMP13, significantly increased, whereas the genes related to senescence SIRT6 and Cav1 decreased in Plcg1^−/−^ chondrocytes compared with Plcg1^f/f^ chondrocytes. These findings implicated the involvement of PLCγ1 in the regulation of chondrocyte senescence, with its inhibition leading to a pronounced senescent state. Additionally, chondrocyte‐specific Plcg1 deficiency, beginning 6‐week post‐induction and continuing through 8, 10 and 12 weeks, has been shown to accelerate the ageing process in both articular cartilage and subchondral bone, as evidenced by elevated expression levels of ageing‐related gene proteins, decreased ratio of HC/HC + CC, diminished levels of COL2 and Agg, and abnormal bone formation parameters in Plcg1^flox/flox^; Col2a1‐CreERT mice. Collectively, our findings not only showed the role of PLCγ1 as a regulator in chondrocyte senescence but also indicated its potential impact on the negative acceleration of the ageing process. However, earlier research has linked PLCγ1 to OA pathogenesis and PLCγ1 inhibition exhibits a chondroprotective impact on IL‐1β‐treated or OA chondrocytes.^6^, ^7^, ^8^, ^9^, ^10^, ^11^ These contradictory findings suggested that the causal role of PLCγ1 in age‐related OA did not terminate and PLCγ1 may play a unique role in the ageing process and OA pathogenesis. This complexity is paralleled in the research on IL‐6, a primary cytokine linked to ageing and age‐related disease.^3^, ^26^, ^27^, ^28^ IL‐6 levels in systemic circulation increase with age.^3^, ^26^ Meanwhile, IL‐6 is an important factor in OA,^27^, ^28^ with its inhibition being an effective therapy approved for clinical use in the pathogenesis of OA.^29^ However, the deletion of IL‐6 in male mice has paradoxically led to more severe (rather than less severe) age‐related OA.^30^ This indicates that the causal relationship between IL‐6 and age‐related OA remains elusive and that additional mediators might be involved in IL‐6 execution. Moreover, given that multiple signal molecules change with both age and OA, it is improbable that the link between age and OA could be attributed to a single factor. For example, inflammaging may indicate more than just an increase in proinflammatory markers but also a delicate balance between them.^3^ Therefore, the discrepancy effects of PLCγ1 in age and OA suggest that other signal molecules may be influencing PLCγ1 deficiency‐dependent chondrocyte senescence. This, in turn, may account for the observed differences in the impact of PLCγ1 on the ageing process versus OA pathogenesis.

Additionally, consistent with previous studies that the administration of d‐Gal decreases frame and femur volume and increases porosity and frame density compared to the control group,^23^, ^31^ we found that d‐Gal induced subchondral bone defects in the femur and tibia. Remarkably, our findings showed that d‐Gal was capable of inducing rat or mouse chondrocytes to exhibit senescent characteristics, including an increase in the number of SA‐β‐Gal positive cells, levels of IL‐8, MMP13, P16, P21 and P53, and a decrease in the levels of SIRT6, Cav1, COL2 and Agg, even at varying dosages of d‐Gal. Meanwhile, elevated P16, P21 and P53 levels and decreased COL2 and Agg levels were observed in the articular cartilage of C57BL/6 mice intraperitoneally injected with d‐Gal. Therefore, d‐Gal could be an effective inducer for developing in vitro and in vivo models of chondrocyte senescence and articular cartilage ageing. The articular cartilage of C57BL/6 mice intraperitoneally injected with d‐Gal revealed no obvious damage compared with the control mice, with the exception of a thinner HC layer, more likely due to the intraperitoneal administration route of d‐Gal, which reduced the effect of d‐Gal on articular cartilage. Another possibility is that articular cartilage first exhibited ageing rather than OA characteristics in response to d‐Gal injection.

Our study is subject to several notable limitations. Firstly, when an 8‐week regimen of d‐Gal treatment is enough to induce senescence in chondrocytes, ageing in articular cartilage and abnormalities in subchondral bone, a comprehensive understanding of OA in articular cartilage necessitates long‐term observation beyond this period. Secondly, although the inhibition of PLCγ1 was observed to accelerate chondrocyte senescence, the underlying mechanism remains elusive. Lastly, we did not assess the long‐term alterations in articular cartilage and subchondral bone that may occur following chondrocyte‐specific Plcg1 deficiency over extended periods of survival.

## AUTHOR CONTRIBUTIONS

**Qiubo Zhao:** Data curation (lead); formal analysis (lead); investigation (lead); methodology (lead). **Xiaolei Chen:** Funding acquisition (equal); methodology (equal); validation (equal). **Ning Qu:** Supervision (lead). **Jinhua Qiu:** Supervision (equal). **Bing Zhang:** Conceptualization (equal); project administration (lead); writing – original draft (lead). **Chun Xia:** Conceptualization (lead); funding acquisition (lead); writing – review and editing (lead).

## CONFLICT OF INTEREST STATEMENT

The authors have declared no competing interests exist.

## Supporting information


Figure S1.

Figure S2.

Figure S3.

Figure S4.

Figure S5.

Figure S6.

Figure S7.

Figure S8.

Figure S9.

Figure S10.

## Data Availability

The relevant data in this study are available from the corresponding author upon reasonable request.
