# Supplementary figures and images for: PLCγ1 deficiency in chondrocytes accelerates the age‐related changes in articular cartilage and subchondral bone
Source: J Cell Mol Med. 2024 Aug 19;28(16):e70027. doi: 10.1111/jcmm.70027 (PMC11332598; doi:10.1111/jcmm.70027)

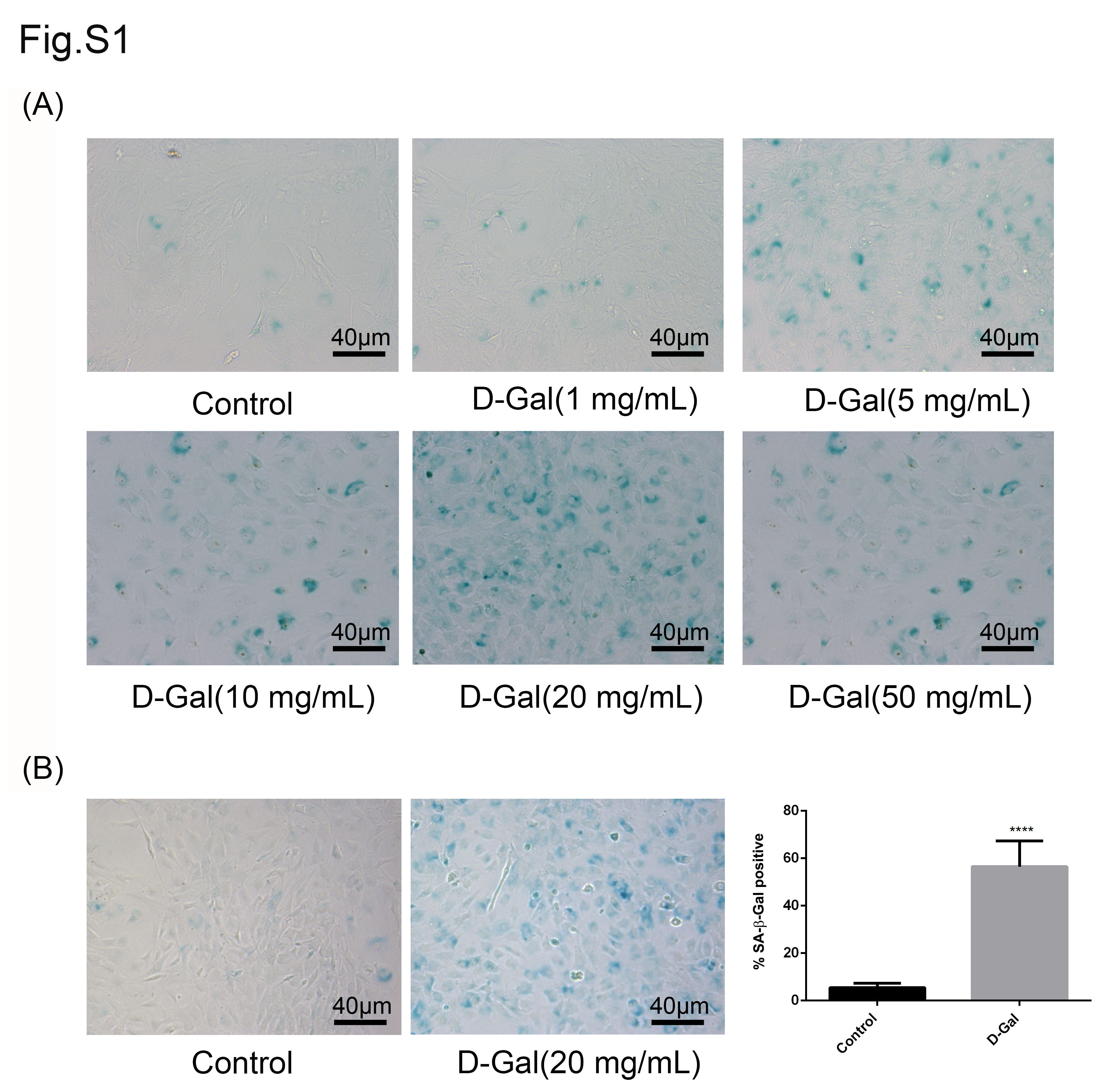

Supplement: Supplementary file 1 — Figure S1. [file JCMM-28-e70027-s010.tif]

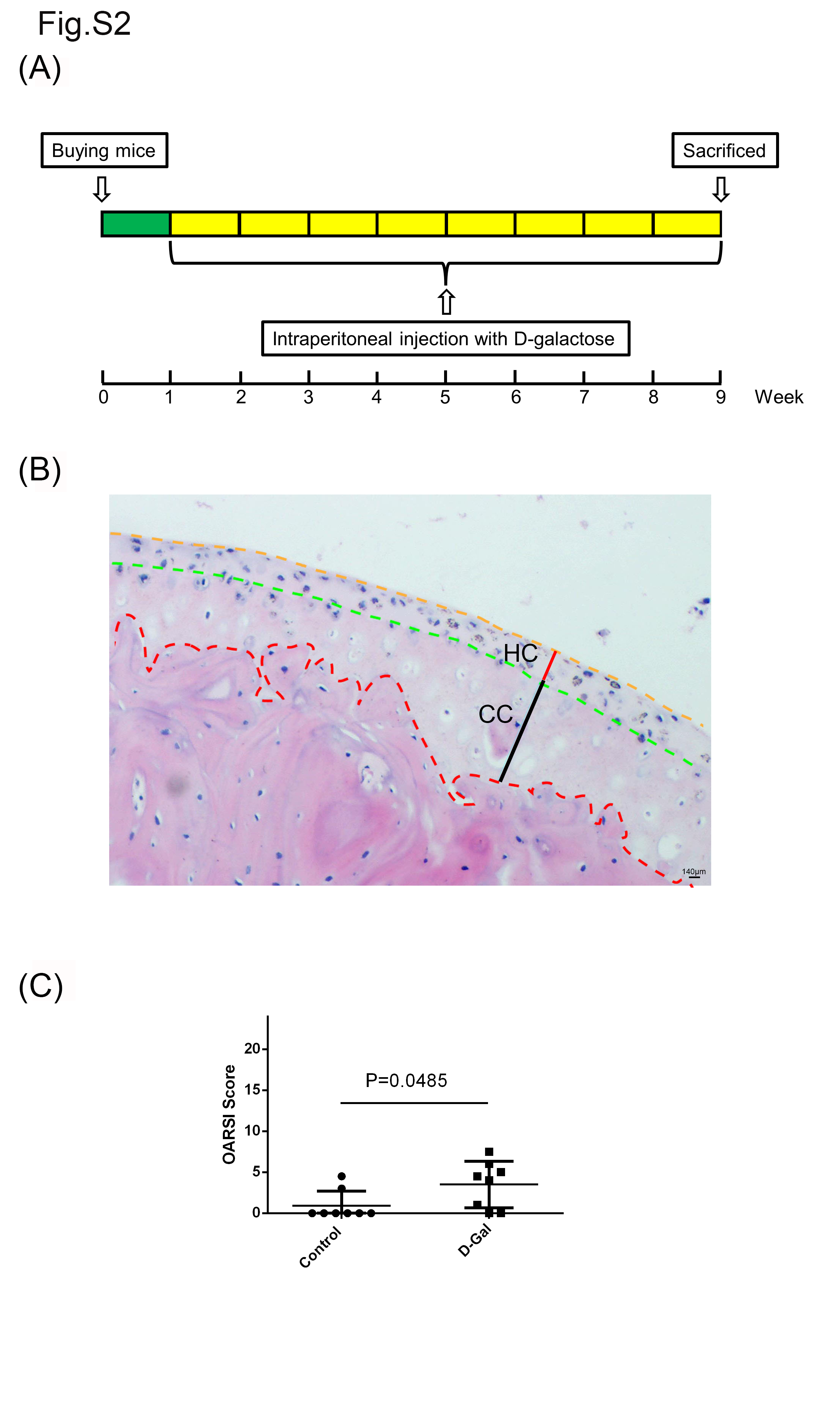

Supplement: Supplementary file 2 — Figure S2. [file JCMM-28-e70027-s007.tif]

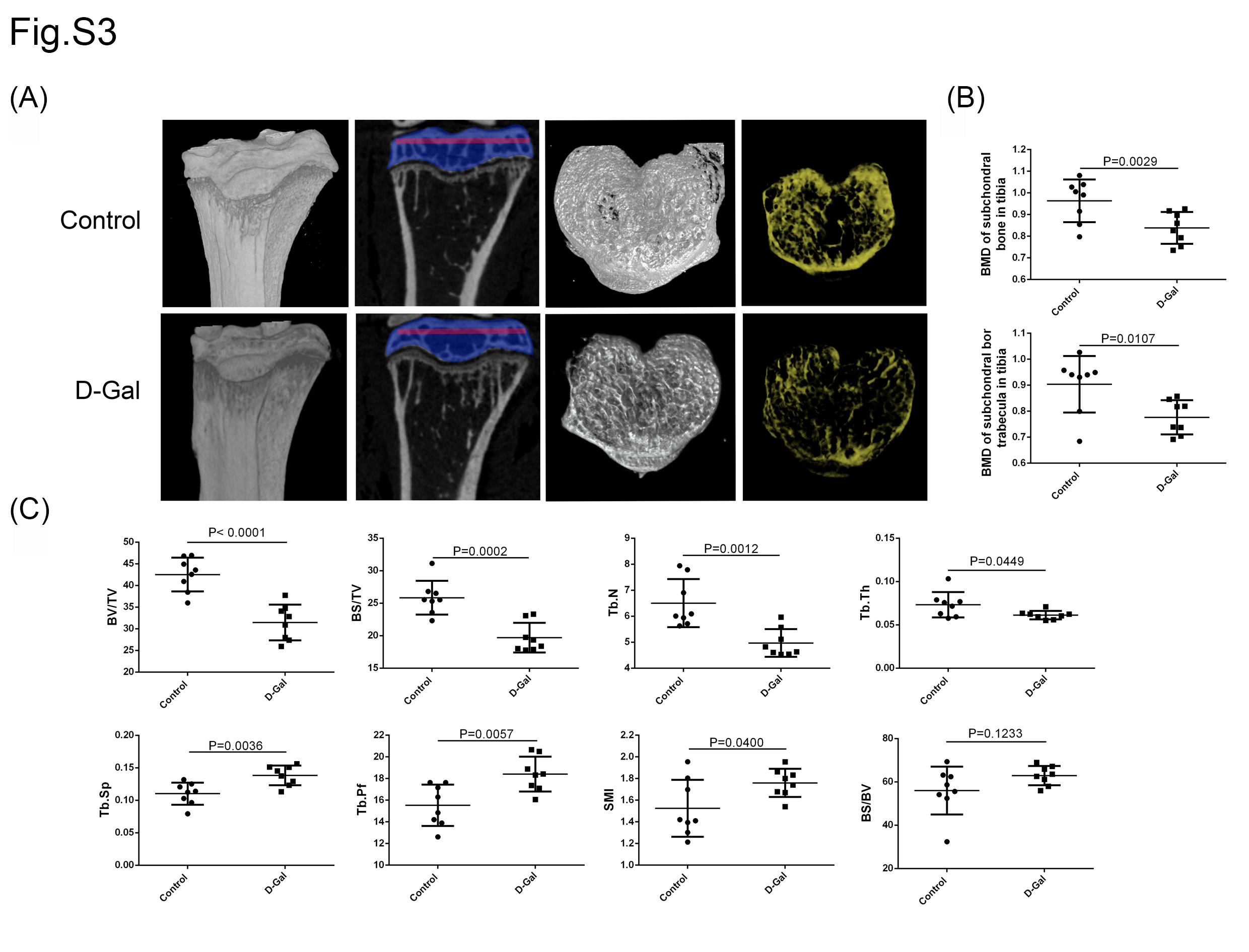

Supplement: Supplementary file 3 — Figure S3. [file JCMM-28-e70027-s009.tif]

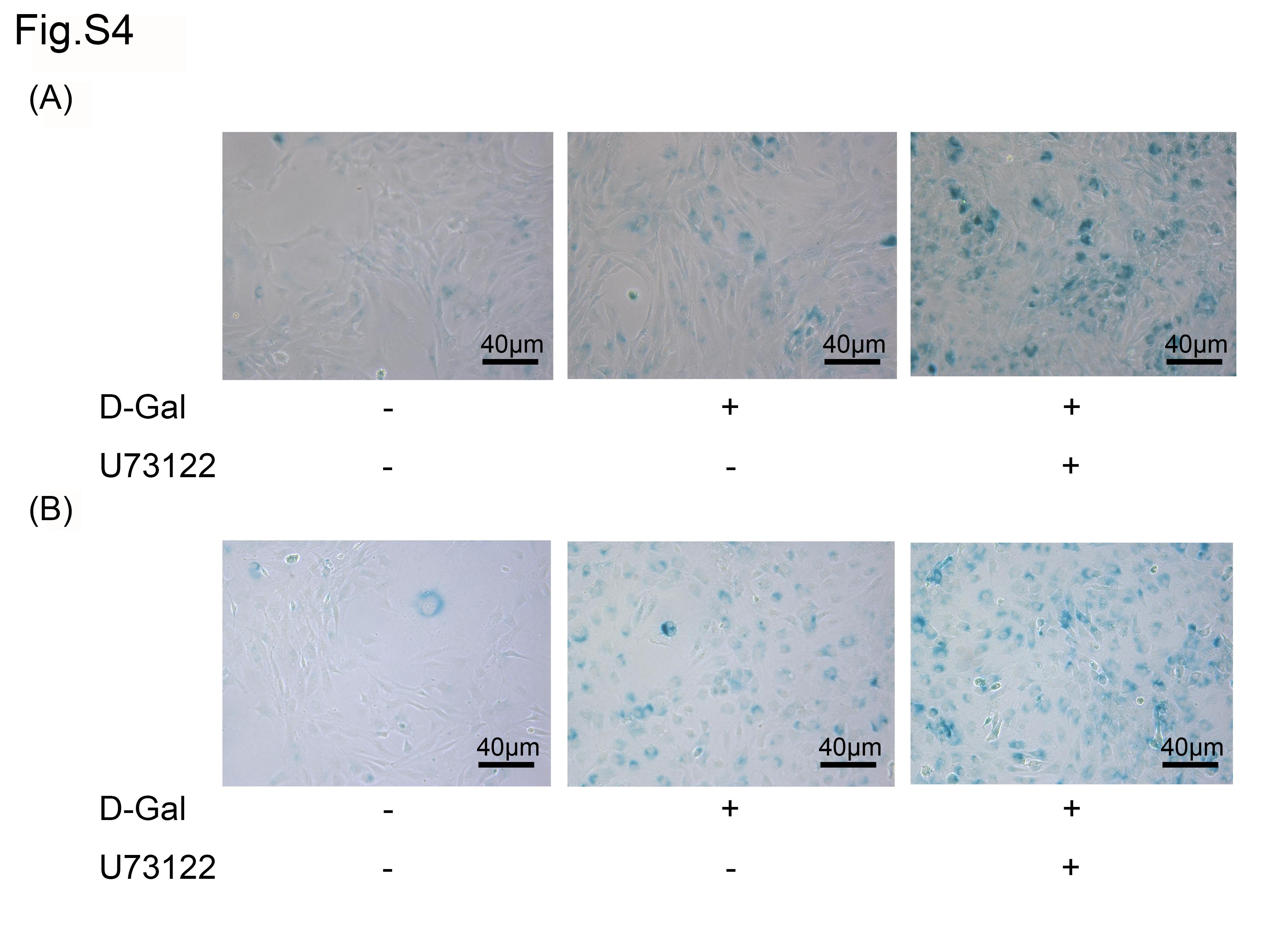

Supplement: Supplementary file 4 — Figure S4. [file JCMM-28-e70027-s006.tif]

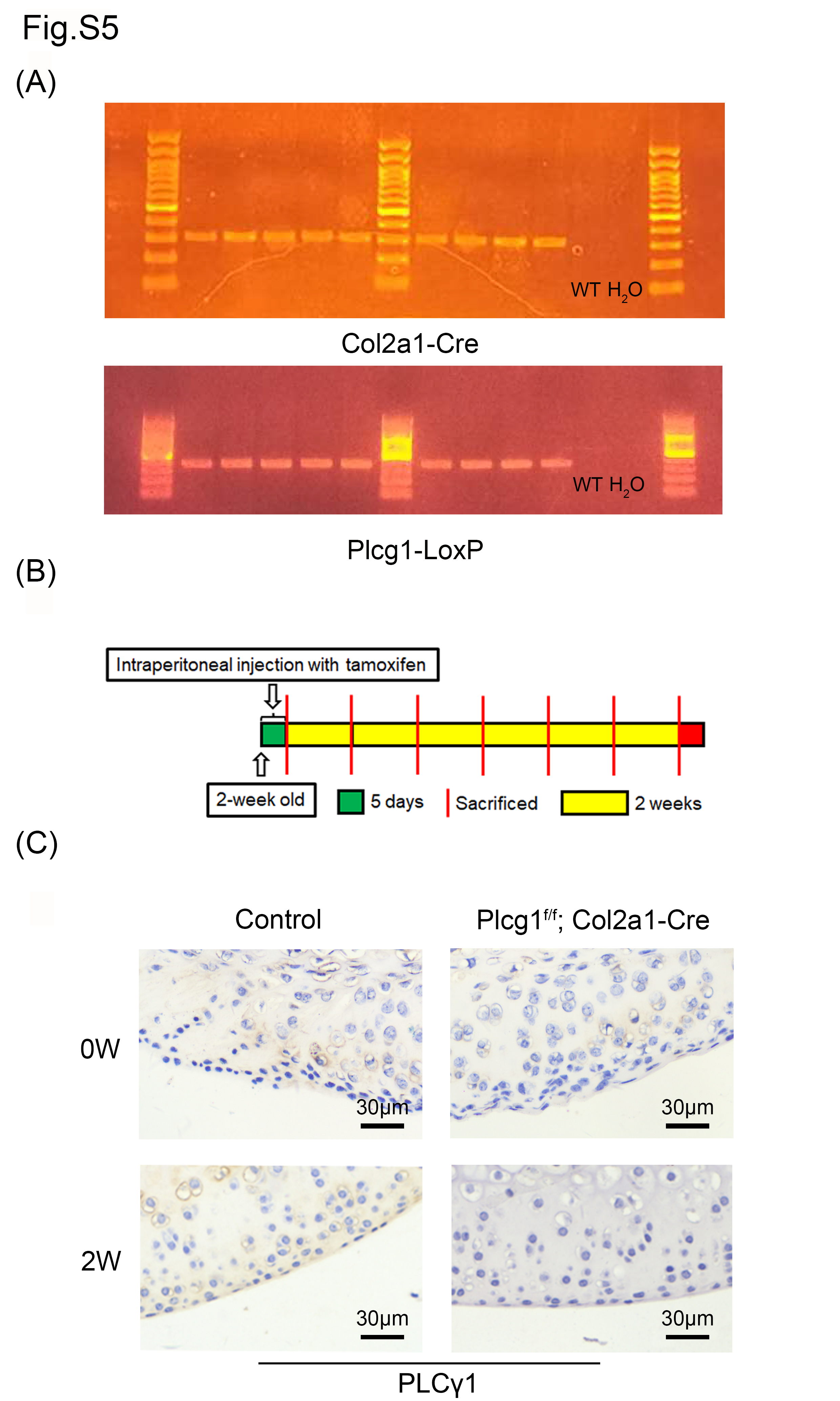

Supplement: Supplementary file 5 — Figure S5. [file JCMM-28-e70027-s004.tif]

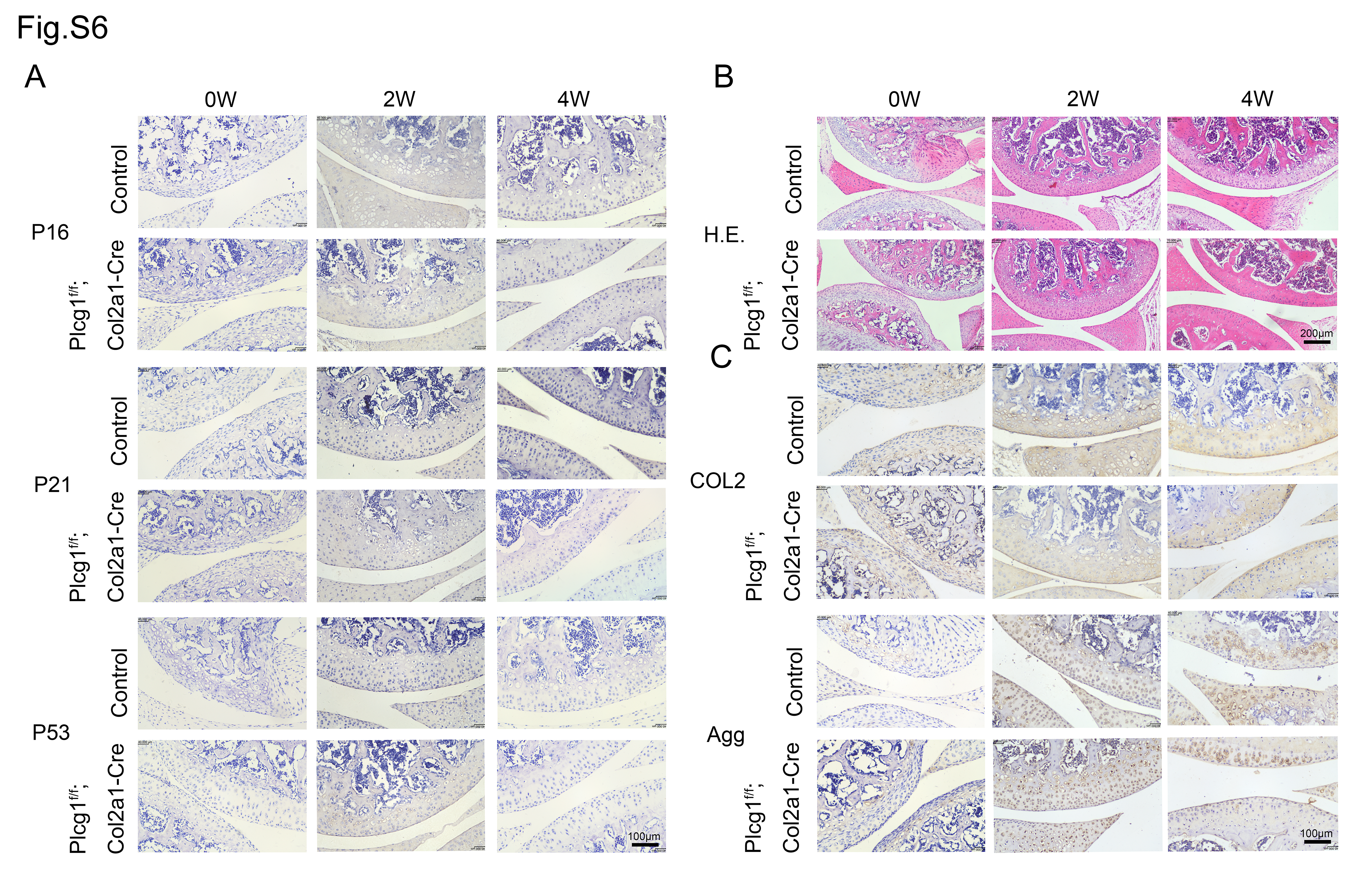

Supplement: Supplementary file 6 — Figure S6. [file JCMM-28-e70027-s003.tif]

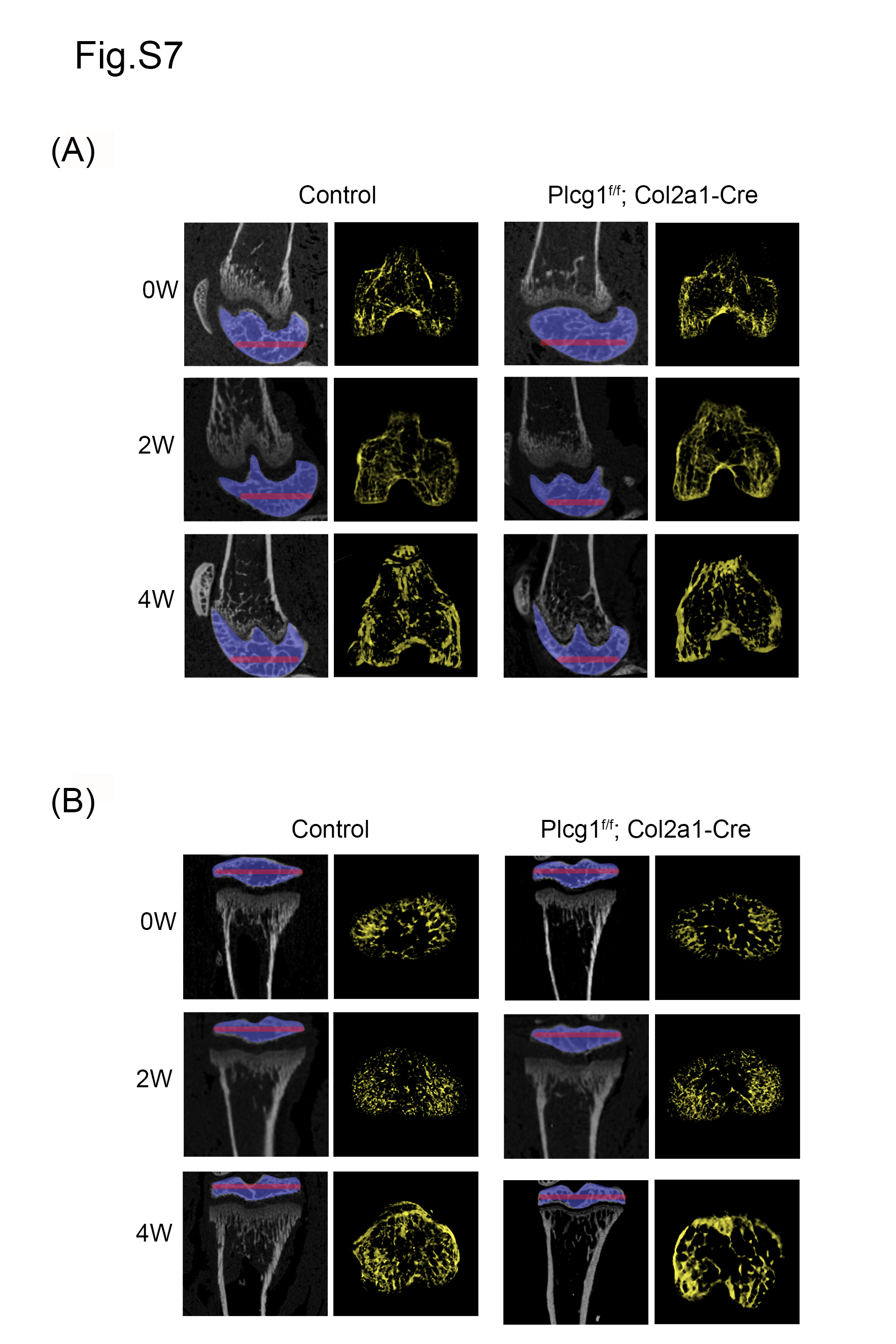

Supplement: Supplementary file 7 — Figure S7. [file JCMM-28-e70027-s008.tif]

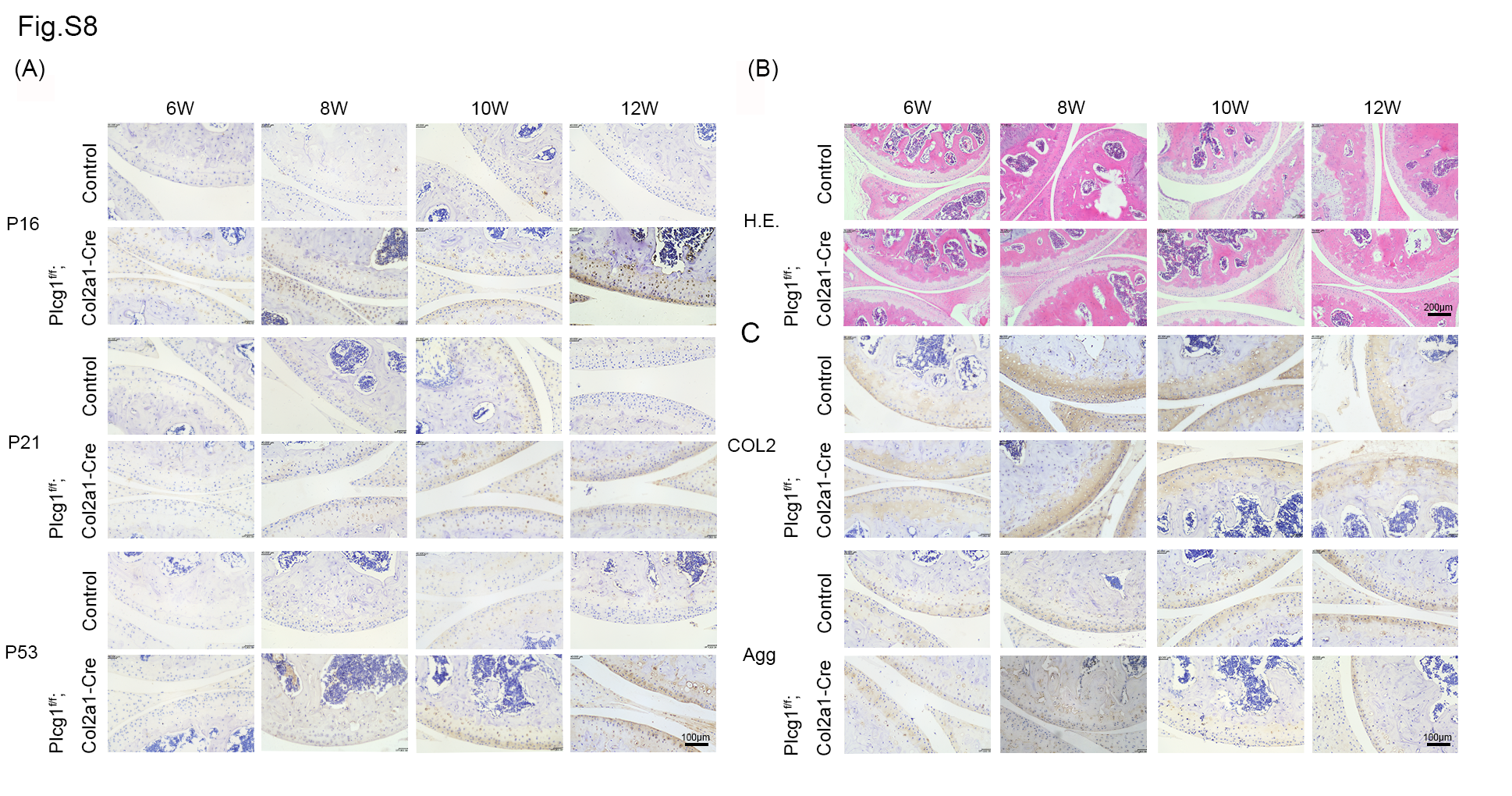

Supplement: Supplementary file 8 — Figure S8. [file JCMM-28-e70027-s002.tif]

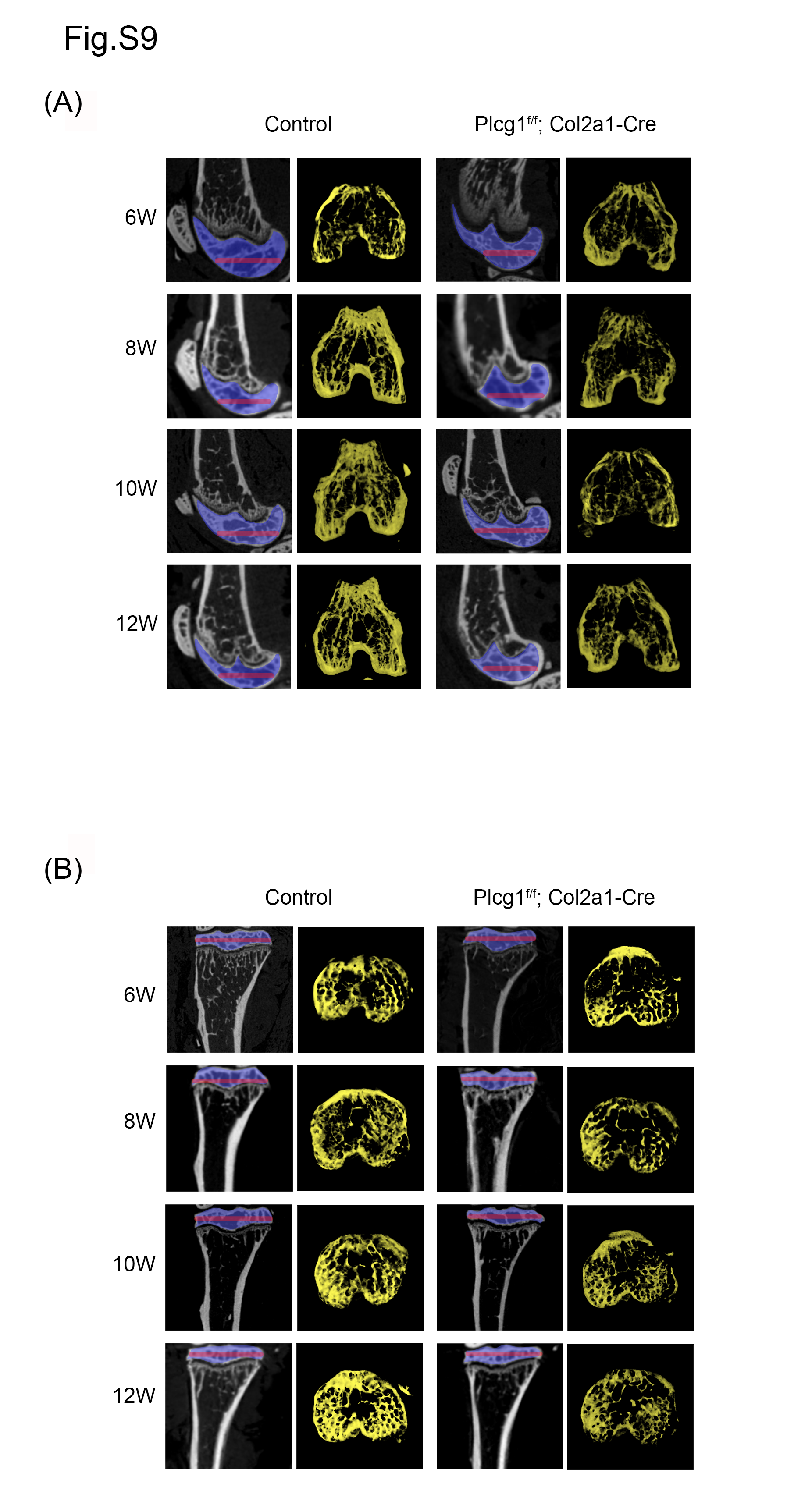

Supplement: Supplementary file 9 — Figure S9. [file JCMM-28-e70027-s005.tif]

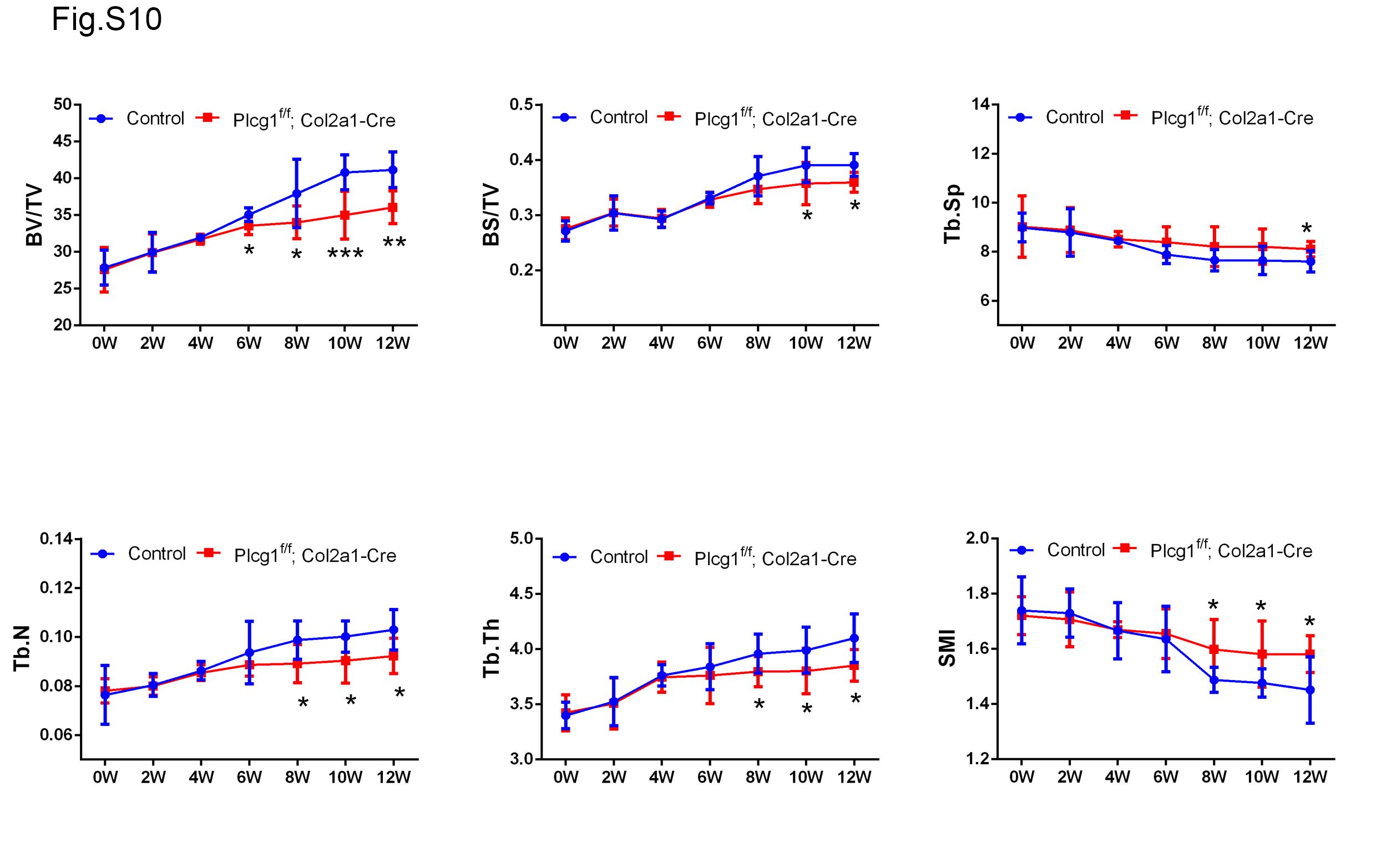

Supplement: Supplementary file 10 — Figure S10. [file JCMM-28-e70027-s001.tif]
